# Why breast muscle satellite cell heterogeneity is an issue of importance for the poultry industry: An opinion paper

**DOI:** 10.3389/fphys.2022.987883

**Published:** 2022-08-15

**Authors:** Sandra G. Velleman

**Affiliations:** Department of Animal Sciences, The Ohio State University, Columbus, OH, United States

**Keywords:** Poultry, Muscle, satellite cells, heterogeneity, Muscle fiber

## Introduction

Skeletal muscle development and growth is unique in that it is characterized by two populations of cells, the myoblasts and a mononucleated adult stem cell termed satellite cells. Satellite cells are stem cells due to their asymmetric division in which one daughter cell will continue myogenesis and the other will replenish the muscle precursor cell reservoir. Skeletal muscle growth in poultry occurs through the formation of myofibers or hyperplasia of myoblasts with myoblasts fusing from to multinucleated myotubes that mature into muscle fibers and muscle fiber bundles. At hatch, myofiber formation is complete (Smith, 1963) and subsequent growth is through the donation of nuclei into existing fibers from satellite cells resulting in the enlargement of fibers through hypertrophic growth (Moss and LeBlond, 1971). The repair and regeneration of muscle fibers from damage is also due to satellite cells.

## Satellite cell heterogeneity

The satellite cell is a mononuclear cell closely positioned adjacent to the sarcolemma of the myofiber (Mauro, 1961). Satellite cells are located on the periphery of the myofibers which resulted in the name satellite cells. The satellite cell contains primarily nuclear material, including a small amount of cytoplasm and only a few organelles, such as a mitochondria or Golgi apparatus. At hatch, satellite cells compose 30% of the total myonuclei of muscle. At the end of the growth period, satellite cell numbers diminish to 1–5% of the total nuclei and their proliferative capacity decreases (Hawke and Garry, 2001). Once growth is complete, the satellite cells will become quiescent (Schultz et al., 1978) and reside in their niche environment until activated to regenerate damaged myofibers. In heavy-weight meat type chickens, satellite cell myogenic activity persists at a high level for the first 8 days posthatch and peaks on day 3 of posthatch age (Halevy et al., 2000).

Satellite cells are not a homogenous population of cells within a muscle. Although satellite cells are commonly referred to as one population of cells, there is heterogeneity of satellite cells within a muscle indicating that there is more than one population. The lack of homogeneity of satellite cells was initially reported by Schultz and Lipton (1982) who showed that proliferation potential of satellite cells was age dependent. Satellite cell heterogeneity can be characterized by satellite cells from different muscle fiber types expressing the genes of the fiber type it originated from (Feldman and Stockdale, 1991; Lagord et al., 1998; Huang et al., 2006; Manzano et al., 2011), and differing rates of proliferation in satellite cells isolated from the same muscle (McFarland et al., 1995; Schultz, 1996). Heterogeneity of satellite cells exists in a single fiber-type muscle like the turkey and chicken pectoralis major muscle that contains homogenous Type IIb fibers. McFarland et al. (1995) isolated 73 different individual satellite cells from the same pectoralis major muscle of a 6 week old tom turkey. The isolated cells were expanded and studied for proliferation, differentiation, and growth factor responsiveness. A range of fast growing to slow growing satellite cells were obtained. The fastest growing satellite cell reached 65% confluency in 17 days whereas the slowest growing satellite cell took 30 days. Differentiation was affected in a similar manner to proliferation. Muscle mass accretion is a result of satellite cell-mediated growth and these functional differences in satellite cells will impact muscle growth and subsequent meat quality of the breast muscle. Furthermore, Xu et al. (2022a, b) showed satellite cells, based on their growth potential, have differences in their regulation of signal transductions pathways like mechanistic target of rapamycin (mTOR) and wingless type mouse mammary tumor virus integration site family/planar cell polarity (Wnt/PCP) pathways. Mechanistic target of rapamycin signal transduction is a key pathway involved in muscle fiber hypertrophy and Wnt/PCP is involved with satellite cell proliferation and differentiation, and lipid synthesis.

## Importance of satellite cell biology for breast muscle growth and regeneration, and myopathies

For the commercial poultry industry, focus needs to be placed on how selection for increased breast muscle yield has altered the biology of satellite cells. These changes in satellite cell biology will alter the morphological structure of the muscle and are associated with myopathies that are detrimental to meat quality like Wooden Breast and Spaghetti Meat. Meat quality reflects both the cell biology and biochemistry of the breast muscle. The reason that attention needs to be focused on satellite cells is that they are critical to both posthatch myofiber growth and regeneration of the myofibers in response to injury. With regard to hypertrophic myofiber growth, cross sectional area of a breast muscle myofiber has increased 3 to 5 times resulting in giant fibers (Dransfield and Sosnicki, 1999). The presence of giant fibers limits endomysial and perimysial spacing between individual myofibers and muscle fiber bundles. The consequences of reducing connective tissue area include myofibers and muscle fiber bundles making contact initiating muscle injury and satellite cell mediated repair (Velleman et al., 2003), and reducing available space for capillary supply to the muscle which is required for satellite cell activity (Velleman, 2015). A regenerated muscle fiber should be identical to the originating fiber. For repair to occur, satellite cell activation from a quiescent state to enter the cell cycle is required for proliferation and differentiation to regenerate the damaged muscle fiber. A required element for satellite cell activation is the proximity of satellite cells to circulatory supply in the satellite cell niche. Christov et al. (2007) and Rhoads et al. (2009) demonstrated that direct communication between the satellite cells and vascular system is required. The regeneration of damaged muscle fibers requires satellite cells to be near capillaries. Satellite cells must be within 21 μm of capillaries in humans to actively regenerate a muscle fiber (Christov et al., 2007). In Wooden Breast-affected breast muscle, satellite cell repair and regeneration of necrotic muscle fibers has been negatively impacted leading to meat quality downgrades (Clark and Velleman, 2017; Velleman et al., 2018). The end result of satellite cell-mediated regeneration is the restoration of the damaged muscle fibers back to their original state which does not occur in the Wooden Breast myopathy. Wooden Breast affected muscle is characterized by a high percentage of smaller diameter myofibers with disorganized contractile sarcomeres (Clark and Velleman, 2017; Velleman et al., 2018). Despite the smaller diameter myofibers with a high degree of myofibril sarcomeric disorganization, satellite cell-mediated regeneration is activated as supported by the increased expression of myogenic transcriptional regulatory factors modulating satellite cell proliferation and differentiation (Velleman and Clark, 2015), but the repair process is still inhibited. Since selection for breast muscle yield in most meat-type commercial broiler lines has resulted in an increase in myofiber diameter through hypertrophic growth, it is likely that the decrease in circulatory supply to the muscle, which is necessary for satellite cell-mediated regeneration, is associated with suppression of myofiber regeneration. These findings suggest that selection for increased breast muscle growth has altered the populations of satellite cells found in the broiler breast muscle to ones with reduced regenerative capabilities. As part of selection for breast muscle mass accretion, the functional characteristics of the satellite cells for myofiber growth through hypertrophy and their activation to regenerate damaged myofibers needs to be assessed to maintain appropriate muscle structure and avoid conditions negatively impacting meat quality.

Since the satellite cells are a stem cell, they can also differentiate into cellular fates other than muscle especially fat. During proliferation, some daughter cells self-renew to maintain the satellite cell pool (Kuang et al., 2007), others follow a myogenic pathway (Kuang et al., 2007), while some will spontaneously convert to an adipogenic cellular lineage (Rossi et al., 2010). Growth selection has likely altered the breast muscle satellite cell population to one with appropriate stimuli that coverts to an adipogenic cellular fate instead of terminally differentiating into muscle. It has been shown that satellite cells during their period of peak mitotic activity are responsive to extrinsic stimuli like nutrition (Velleman et al., 2014a) and temperature (Xu et al., 2021a). Feed restrictions during the peak period of mitotic activity, the first week after hatch, result in pectoralis major muscle satellite cells synthesizing lipid which becomes intramuscular fat depots in the breast muscle (Velleman et al., 2014b) that are similar to intramuscular marbling fat depots observed in bovine meat (Smith and Johnson, 2014). Increased intramuscular fat will negatively impact the protein to fat ratio in the breast muscle meat product. This change in the protein to fat ratio is converse to poultry breast meat being sold as a low-fat protein choice. Thermal stress especially heat also affects the cellular fate of satellite cells (Clark et al., 2017; Xu et al., 2021b). Satellite cells are most sensitive to temperature when they are proliferating and growth selection has increased the thermal sensitivity of satellite cells to synthesize lipid (Halevy, 2020; Xu et al., 2021b).

Although even within a uniform muscle fiber type like the poultry breast muscle, heterogeneity within the satellite cell populations composing a muscle like the breast muscle has likely been altered by selection for breast muscle yield. Satellite cells dynamically express cell surface receptors (Yin et al., 2013) involved with key processes, including but not limited to, cell migration, growth factor responsiveness, and proliferation. Thus, selection for breast muscle yield has likely altered the balance between the different populations of satellite cells which could result as already demonstrated by Xu et al. (2021b) in satellite cells from growth selected meat-type turkeys being more prone to transdifferentiate to an adipogenic fate or as in broilers a decreased potential of satellite cells to activate from a quiescent state to regenerate damaged myofibers (Velleman et al., 2018).

## Discussion

Heavy weight fast growing meat-type poultry is characterized by excessive myofiber hypertrophic growth mediated by the satellite cells due to growth selection occurring during the posthatch period of satellite cell-mediated muscle mass accretion. The large diameter myofibers reduce connective tissue spacing in the endomysial and perimysial areas resulting in muscle fibers and fiber bundles making contact and initiating fiber degeneration (Wilson et al., 1990; Velleman et al., 2003). The juxtaposed position of the muscle fibers and bundles also limits spacing for circulatory supply which is necessary for satellite cell activity to repair muscle fiber damage. Additionally, as shown in previous studies satellite cells from modern heavy weight fast growing meat-type broilers have reduced regeneration and repair of damaged myofibers (Velleman et al., 2018), and in both broilers and turkeys the satellite cells are more prone to transdifferentiate to an adipogenic lineage rather than follow a muscle terminal differentiation cellular fate (Clark and Velleman, 2017; Xu et al., 2021b). Since satellite cells are critical to the development, growth, and regeneration of muscle, management strategies must evolve to include the cellular biology of satellite cells including population heterogeneity and their effect on the morphological structure of the breast muscle. Strategies encompassing satellite cell biology will likely significantly decrease meat quality downgrades from breast muscle myopathies.
